# Extracellular vesicle-mediated cell–cell communication in keloids and hypertrophic scars: mechanisms, methodological caveats, and therapeutic perspectives

**DOI:** 10.3389/fcell.2026.1818463

**Published:** 2026-06-01

**Authors:** Bo Lu, Junjie Jin, Wenyan Jin, Xinghua Yuan, Zhehu Jin

**Affiliations:** 1 Department of Dermatology, Yanbian University Hospital, Yanbian University Medical College, Jilin, China; 2 Department of Dermatology, Air Force Medical Center, Air Force Medical University, Beijing, China

**Keywords:** exosomes, fibrosis, hypertrophic scars, intercellular communication, keloids

## Abstract

Pathological scarring, including keloids and hypertrophic scars, remains a major clinical challenge characterized by persistent fibroblast activation, excessive extracellular matrix deposition, chronic inflammation, and aberrant tissue remodeling. Although current treatments have improved management, high recurrence rates and variable outcomes indicate that the underlying mechanisms remain incompletely understood. Extracellular vesicles (EVs), especially small extracellular vesicles (sEVs), are increasingly recognized as mediators of intercellular communication in fibrotic skin disorders. However, according to MISEV recommendations, the term “exosome” should be used cautiously unless endosomal origin is specifically demonstrated. In pathological scars, EV-/sEV-mediated signaling has been implicated in fibroblast activation, immune dysregulation, angiogenic imbalance, and extracellular matrix remodeling through cargo transfer among fibroblasts, keratinocytes, endothelial cells, immune cells, and mesenchymal stromal/stem cells. Reported downstream pathways include TGF-β/Smad, Wnt/β-catenin, PI3K/Akt, and NF-κB. However, the strength of evidence varies substantially, as many conclusions are based on *in vitro* studies or correlative omics analyses rather than direct causal experiments. In addition, differences in EV isolation and characterization methods may significantly affect reported cargo profiles and functional outcomes. This review critically summarizes EV-/sEV-mediated communication in keloids and hypertrophic scars, emphasizing vesicle nomenclature, methodological rigor, cell-specific signaling, and the distinction between causal and associative evidence. We also discuss translational challenges, including large-scale production, batch heterogeneity, storage stability, biodistribution, targeting, safety, and regulatory considerations.

## 1 Introduction

Pathological scarring, including keloids and hypertrophic scars, is a fibroproliferative outcome of aberrant wound healing characterized by persistent inflammation, excessive extracellular matrix (ECM) deposition, abnormal tissue stiffness, and incomplete resolution of repair responses (Limandjaja et al., 2020). Clinically, keloids extend beyond the original wound margins and rarely regress, whereas hypertrophic scars remain confined to the site of injury and may partially regress over time (Ogawa, 2017). In addition to cosmetic disfigurement, both lesions may cause pain, pruritus, contracture, and substantial psychosocial burden (Berman et al., 2017). Although current treatments, such as surgical excision, intralesional corticosteroids, radiotherapy, and laser-based approaches, can improve some lesions, recurrence and variable therapeutic responses remain major limitations (Ud-Din and Bayat, 2014). These challenges underscore the need for a more precise understanding of the cellular and molecular mechanisms that drive pathological scar formation and persistence.

Pathological scars are increasingly recognized as microenvironment-driven disorders involving dynamic interactions among fibroblasts, keratinocytes, endothelial cells, immune cells, and mesenchymal stromal/stem cells (Zhang et al., 2023a). Soluble mediators such as cytokines, chemokines, and growth factors contribute to this crosstalk, but they do not fully explain the spatial and sustained nature of fibrotic signaling within scar tissue (Kim and Kim, 2024; Narauskaitė et al., 2021). Extracellular vesicles (EVs), particularly small extracellular vesicles (sEVs), have therefore attracted growing attention as additional mediators of intercellular communication. By transporting proteins, lipids, and nucleic acids—including microRNAs, long non-coding RNAs, and circular RNAs—EVs may influence fibroblast phenotype, immune regulation, angiogenesis, and ECM remodeling in scar tissue (Prasai et al., 2022; Ludwig and Giebel, 2012; Prasai et al., 2022; Qin et al., 2023).

Accumulating studies suggest that EV-associated cargos may modulate key fibrotic and inflammatory pathways, including TGF-β/Smad, Wnt/β-catenin, PI3K/Akt, and NF-κB, thereby contributing to scar initiation, progression, and persistence (Wang et al., 2022a; Yang et al., 2023). In many reports, the term “exosome” is used broadly even when endosomal origin has not been demonstrated, and differences in vesicle isolation and characterization methods may substantially influence the reported cargo composition and biological effects. (Qin et al., 2020; Aljohani, 2025). In addition, much of the available evidence is derived from cell culture experiments, animal models, or omics-based association studies, whereas direct causal validation and clinical evidence remain limited.

In this review, we critically examine EV-/sEV-mediated intercellular communication in keloids and hypertrophic scars. We emphasize three issues that are often underappreciated in the field: first, the need to align vesicle nomenclature and characterization with current MISEV recommendations; second, the importance of distinguishing direct causal evidence from correlative cargo profiling or pathway enrichment analyses; and third, the translational challenges that currently limit therapeutic application, including manufacturing scalability, batch variability, storage stability, biodistribution, targeting, safety, and regulatory considerations. We also organize the literature by cellular source and biological context to clarify how EV cargo may influence fibroblast activation, immune responses, and ECM remodeling in pathological scar formation.

## 2 Extracellular vesicles: biogenesis, cargo, and functional roles

### 2.1 Biogenesis and secretion pathway

Exosomes are typically defined as small extracellular vesicles of endosomal origin generated through the multivesicular body (MVB) pathway. During this process, inward budding of late endosomal membranes forms intraluminal vesicles (ILVs), which are subsequently released into the extracellular space upon fusion of MVBs with the plasma membrane. Early endosomes mature into MVBs, where ILV formation is coupled to cargo sorting (Han et al., 2022). The endosomal sorting complex required for transport (ESCRT) machinery plays a central role in this process by regulating cargo recognition, membrane budding, and vesicle scission, although ESCRT-independent mechanisms have also been described (Tschuschke et al., 2020; Marie et al., 2023). Ceramide generated by neutral sphingomyelinase 2 (nSMase2) may facilitate membrane curvature and ILV formation (Choezom and Gross, 2022). In addition, Rab GTPases (e.g., Rab27a/b, Rab11, and Rab35) and SNARE proteins regulate vesicle trafficking, docking, and membrane fusion, thereby influencing vesicle secretion efficiency across different cellular contexts (Blanc and Vidal, 2018).

### 2.2 Cargo composition

Extracellular vesicles carry diverse molecular cargos that reflect the physiological and pathological state of donor cells. These cargos include nucleic acids (miRNAs, lncRNAs, circRNAs, mRNAs, and occasionally DNA fragments), proteins, lipids, and metabolites, all of which may influence recipient cell behavior (Chen et al., 2024; Kalluri and LeBleu, 2020; Nail et al., 2023). RNA loading into EVs appears to be selectively regulated rather than random, with RNA-binding proteins and sequence motifs contributing to cargo enrichment (Lee et al., 2024). EVs also transport proteins such as tetraspanins (CD9, CD63, and CD81), heat-shock proteins, signaling molecules, and enzymes, which may directly modulate signaling pathways in recipient cells (Kalluri and LeBleu, 2020). Additionally, lipid components, including cholesterol, sphingomyelin, and ceramides, contribute to vesicle stability and membrane fusion dynamics (Chen et al., 2024; Kalluri and LeBleu, 2020; Nail et al., 2023).

### 2.3 Mechanisms of uptake and target specificity

After release, EVs interact with recipient cells through receptor–ligand interactions, membrane fusion, or endocytosis (Mathieu et al., 2019). Uptake mechanisms include clathrin-mediated endocytosis, caveolin-dependent endocytosis, lipid raft–associated internalization, and macropinocytosis (Mulcahy et al., 2014). Target specificity is influenced by vesicle surface molecules, including integrins, tetraspanins, and proteoglycans, which facilitate selective binding and uptake (Hoshino et al., 2015). These interactions determine intracellular trafficking routes and functional cargo delivery (Joshi et al., 2020). Importantly, microenvironmental conditions such as hypoxia, inflammation, and mechanical stress may alter EV uptake efficiency and signaling outcomes (Gurunathan et al., 2021). However, most mechanistic insights into EV uptake derive from *in vitro* studies, and *in vivo* targeting specificity remains incompletely understood.

### 2.4 Roles of EVs in tissue repair and fibrosis

EVs are increasingly recognized as regulators of wound healing and fibrosis, mediating communication between immune, epithelial, stromal, and vascular cells (Liu and Su, 2019). During the inflammatory phase, immune cell-derived vesicles may influence macrophage polarization and cytokine balance. For example, M2 macrophage-derived vesicles have been reported to deliver miR-223 and miR-146a, promoting anti-inflammatory responses (Huang et al., 2022; Song et al., 2017). Keratinocyte- and endothelial-derived vesicles may promote re-epithelialization and angiogenesis (Ma et al., 2019).

During the proliferative and remodeling phases, fibroblast- and myofibroblast-derived vesicles may reinforce fibrotic programs in recipient fibroblasts by transferring pro-fibrotic mediators such as TGF-β1, CTGF, and ECM-related transcripts (Li C. et al., 2021; Borges et al., 2013). These vesicles have been associated with increased fibroblast proliferation, α-smooth muscle actin (α-SMA) expression, and collagen I/III deposition, with downstream effects often linked to TGF-β/Smad and PI3K/Akt signaling (Xie and Zeng, 2020). In pathological scarring, such fibroblast-centered EV-mediated communication may contribute to self-amplifying fibrotic loops that limit scar regression (Yu et al., 2024).

However, most of these findings derive from *in vitro* experiments or preclinical models, and in many studies, vesicles were classified as “exosomes” without biogenesis-specific validation. Thus, although the available evidence supports a functional role for EVs in fibrosis, the strength of mechanistic inference remains variable across studies.

By contrast, vesicles derived from mesenchymal stromal/stem cells (MSCs), adipose-derived stem cells (ADSCs), and umbilical cord–derived cells are frequently reported to exert anti-inflammatory and anti-fibrotic effects (Zhou et al., 2023). These vesicles may deliver regulatory miRNAs, including members of the miR-29 family and miR-200b, that suppress pro-fibrotic gene expression, dampen TGF-β–associated signaling, and limit myofibroblast differentiation (Wang et al., 2022b). MSC-derived vesicles have also been associated with enhanced re-epithelialization and angiogenesis through ERK- and STAT3-related pathways (Zhang et al., 2015). In experimental models, such vesicles have been linked to reduced collagen deposition, attenuated inflammation, and improved dermal architecture (Wang et al., 2017). Nevertheless, these observations are still largely based on preclinical evidence, and important translational issues—including methodological heterogeneity, production consistency, storage stability, and *in vivo* targeting—remain unresolved.

EV-mediated crosstalk between fibroblasts and immune cells may further shape fibrogenesis (Di et al., 2024). Macrophage-derived vesicles, for example, may promote or restrain fibrosis depending on polarization state: M1-associated vesicles are generally linked to amplification of inflammatory signaling and fibroblast activation, whereas M2-associated vesicles are more often associated with pro-resolution remodeling (Qin et al., 2021). Endothelial-derived vesicles may likewise modulate angiogenesis and vascular integrity, both of which influence scar maturation and remodeling (Yuan et al., 2026). Collectively, these studies suggest that EV-mediated communication contributes to the balance between regenerative repair and persistent fibrosis (Deng et al., 2025). However, it remains important to distinguish between conclusions supported by direct causal experiments and those inferred primarily from correlation, pathway enrichment, or cargo profiling alone.

Dysregulated EV signaling has therefore emerged as a plausible contributor to fibroblast activation, chronic inflammation, and excessive ECM deposition in pathological scars (Zhao et al., 2022). Pro-fibrotic cargos such as miR-21 and lncRNA H19 have been associated with enhanced fibroblast proliferation, reduced apoptotic responsiveness, and increased matrix production in scar-related settings (Li Q. et al., 2021; Qian et al., 2021). In addition, oxidative stress and hypoxia—both common features of fibrotic microenvironments—may alter vesicle release and cargo composition, thereby further amplifying pathogenic intercellular signaling (Qian et al., 2024). Taken together, current evidence supports a role for EVs in wound repair and fibrosis, but also highlights the need for more rigorous classification, standardized methodology, and stronger causal validation in future scar research.

### 2.5 Nomenclature and methodological considerations (MISEV2023)

According to the Minimal Information for Studies of Extracellular Vesicles (MISEV 2023) guidelines, the term “exosome” should be used with caution unless vesicles are specifically demonstrated to originate from endosomal multivesicular bodies. In the pathological scarring literature, many studies have described vesicles as “exosomes” based primarily on size or precipitation-based isolation, often without sufficiently rigorous characterization. Therefore, when biogenesis-specific evidence is unavailable, the term “small extracellular vesicles (sEVs)” is more appropriate. MISEV2023 further emphasizes that EV studies should incorporate rigorous methodological validation, ideally through complementary isolation strategies or orthogonal validation approaches, together with multidimensional characterization. At a minimum, characterization should include particle size distribution analysis (such as NTA or DLS), morphological assessment (such as TEM or SEM), and detection of representative protein markers, including CD9, CD63, CD81, and TSG101. Studies that do not meet these standards should be interpreted cautiously, because differences in isolation methods may substantially affect vesicle purity, molecular composition, and downstream functional readouts.

### 2.6 Methodological variability in EV isolation and its impact on reported cargo and function

Methodological variability in EV isolation can substantially influence the reported vesicle cargo and functional outcomes. Ultracentrifugation, the most commonly used method, remains a conventional approach but may co-isolate protein aggregates, lipoproteins, and other contaminants, thereby complicating downstream interpretation. Precipitation-based commercial kits offer convenience and higher yield; however, they often produce lower-purity preparations and increased contamination with non-vesicular components, which may confound cargo profiling and biological assays. By contrast, size-exclusion chromatography (SEC) generally provides higher purity and better preservation of vesicle integrity, although it may yield lower recovery and often requires additional concentration steps. These methodological differences can lead to variability in detected RNA, protein, and lipid cargo, as well as inconsistencies in reported biological effects. Consequently, differences in isolation strategies should be carefully considered when comparing studies, and conclusions regarding EV-mediated functions in pathological scarring should be interpreted with caution.

## 3 Cellular sources of EVs/sEVs in keloids and hypertrophic scars

Pathological scarring arises from multi-lineage crosstalk within the cutaneous microenvironment, involving fibroblasts, keratinocytes, endothelial cells, immune cells, and mesenchymal stromal/stem cells. In this section, we organize the evidence by cellular source, because vesicle composition and functional output are strongly shaped by donor-cell identity and microenvironmental state. Exosome-enriched small extracellular vesicles released from these populations have been reported to carry distinct regulatory cargo that modulates inflammation, angiogenesis, and ECM remodeling, thereby biasing wound healing toward regeneration or persistent fibrosis.

### 3.1 Fibroblast-derived EVs/sEVs

Fibroblasts are the principal stromal cells responsible for extracellular matrix production and tissue remodeling during wound repair and scar maturation (Hinz, 2007). In pathological scarring, fibroblast-derived EVs have been implicated in reinforcing pro-fibrotic signaling networks. These vesicles reportedly contain TGF-β1, CTGF, ECM-related transcripts, and regulatory miRNAs such as miR-21, miR-199a, and miR-23a (Cui et al., 2022).

Functionally, transfer of fibroblast-derived EVs to recipient fibroblasts or keratinocytes has been associated with activation of TGF-β/Smad and PI3K/Akt pathways, increased α-SMA expression, enhanced collagen deposition, and sustained fibroblast proliferation (Liu et al., 2025). Most of these observations, however, are derived from *in vitro* experiments, with limited *in vivo* validation.

Notably, vesicles derived from keloid fibroblasts have been reported to induce keloid-like phenotypic changes in normal dermal fibroblasts. Furthermore, inhibition of vesicle release through pharmacologic or genetic approaches, such as nSMase2 inhibition or Rab27 knockdown, has attenuated fibroblast activation in some studies, providing stronger support for a causal role of fibroblast-derived EVs in fibrosis (Ostrowski et al., 2010). Nevertheless, such causal evidence remains limited, and further *in vivo* and clinical validation is required.

### 3.2 Keratinocyte-derived EVs/sEVs

Keratinocyte-derived vesicles are important mediators of epidermal–dermal communication and may influence fibroblast behavior during both physiological and pathological wound repair (Ma et al., 2019). Under normal conditions, keratinocyte-derived vesicles have been associated with enhanced fibroblast proliferation and migration, thereby supporting coordinated dermal remodeling (Bo et al., 2022). In pathological scarring, however, keratinocyte dysfunction may alter vesicle cargo composition, shifting it toward a more pro-fibrotic profile that includes dysregulated cytokines, growth factors, and ncRNAs such as miR-23a and miR-130b (Yang and Lv, 2026). These changes have been linked to activation of TGF-β- and Wnt/β-catenin-related pathways in recipient fibroblasts, which may contribute to increased ECM production and persistent fibroblast activation.

In addition to direct stromal effects, keratinocyte-derived vesicles may also influence keratinocyte differentiation and barrier restoration, thereby indirectly shaping inflammatory tone within the wound environment (Lee, 2020). Nevertheless, compared with fibroblast-derived vesicles, the evidence supporting a specific role for keratinocyte-derived EVs in keloids and hypertrophic scars remains more limited and is largely based on inferred signaling relationships rather than direct causal experiments. Accordingly, keratinocyte–fibroblast vesicle crosstalk should be considered an important but still incompletely validated axis in pathological scar biology.

### 3.3 Endothelial cell-derived EVs/sEVs

Endothelial cell-derived vesicles regulate angiogenesis and vascular integrity, both of which are closely linked to wound repair outcomes and scar maturation. These vesicles have been reported to carry pro-angiogenic factors, including miR-126 and other vascular regulators, that promote endothelial proliferation, migration, and tube formation (Liu et al., 2024). In pathological scarring, dysregulated endothelial signaling may contribute to abnormal vascular remodeling, altered oxygen delivery, and chronic inflammatory activation. Consistent with this idea, endothelial-derived vesicles have been associated with immature or hyperpermeable microvascular states that may exacerbate hypoxia and secondarily reinforce fibrogenic signaling (Zhang et al., 2023b).

At the same time, the role of endothelial-derived vesicles is likely context dependent. In some settings, they may support vascular maturation and tissue remodeling, potentially favoring scar resolution rather than fibrosis (Tan et al., 2024). Thus, their effects may depend on donor-cell status, local oxygen tension, and the balance between pro-angiogenic and pro-inflammatory signaling. At present, most conclusions regarding endothelial-derived vesicles in pathological scarring are based on mechanistic plausibility and indirect experimental evidence, rather than on direct demonstration that a defined endothelial vesicle cargo drives a scar-specific phenotype.

### 3.4 Immune cell-derived EVs/sEVs

Immune cell-derived vesicles, particularly those released by macrophages, are increasingly recognized as important regulators of inflammatory and fibrotic responses within scar tissue. Because macrophage polarization states influence vesicle cargo composition, immune cell-derived vesicles may exert either pro-fibrotic or pro-resolution effects. Vesicles associated with M1-like inflammatory states have been linked to cargos such as miR-155 and, in some settings, miR-21, which may amplify fibroblast activation, collagen synthesis, and inflammatory gene expression through pathways including NF-κB-related signaling (Cui et al., 2019). By contrast, vesicles associated with M2-like or pro-resolving states have been reported to contain miR-223, miR-146a, IL-10, and related modulators that may attenuate inflammation and support ECM remodeling (Guiot et al., 2020).

This duality is particularly important in pathological scarring, where persistent inflammatory signaling is thought to contribute to fibroblast dysregulation and refractory fibrosis (Zhu et al., 2021). Beyond macrophages, vesicles derived from T cells, mast cells, and other immune populations may also influence fibroblast behavior, angiogenesis, and inflammatory persistence (Rasaei et al., 2022). However, the current literature remains limited by broad functional categorization of immune-derived vesicles and insufficient cell-specific mechanistic dissection. In many studies, immune cell-derived vesicle effects are inferred from polarization status and downstream phenotype rather than from direct evidence linking a defined vesicle cargo to a validated target pathway. Thus, while immune-derived vesicles are highly relevant to scar pathophysiology, the exact cell source–cargo–pathway relationships require further clarification.

### 3.5 Mesenchymal stem cell (MSC)-derived EVs/sEVs

Vesicles derived from mesenchymal stromal/stem cells have attracted substantial interest because of their reported anti-inflammatory and anti-fibrotic properties in skin repair. MSC-derived vesicles may carry regulatory cargos, including miR-29 family members, miR-200b, lncRNAs, and proteins, that collectively suppress fibroblast activation, dampen TGF-β/Smad signaling, and promote matrix turnover (Zhong et al., 2023). In addition, MSC-derived vesicles have been linked to enhanced angiogenesis and re-epithelialization through ERK- and STAT3-associated pathways in endothelial and epithelial compartments (Yuan et al., 2021). In experimental models of keloids and hypertrophic scars, these vesicles have been associated with reduced collagen deposition, decreased myofibroblast differentiation, and improved dermal architecture (Seo and Jung, 2016; Bojanic et al., 2021).

Among the EV populations discussed in this review, MSC-derived vesicles currently represent the most advanced candidate for therapeutic development. However, the available evidence remains predominantly preclinical, and enthusiasm should be tempered by unresolved translational issues, including manufacturing scale-up, batch-to-batch heterogeneity, storage stability, biodistribution, targeting efficiency, and long-term safety (Hu et al., 2019). Thus, MSC-derived vesicles are best regarded as promising investigational agents rather than established therapeutic tools for pathological scarring.

### 3.6 Comparison of keloids and hypertrophic scars: clinical, histological, and exosome-mediated differences

Keloids and hypertrophic scars share common fibrotic features but differ in clinical behavior and molecular characteristics. Keloids extend beyond wound margins and rarely regress, whereas hypertrophic scars remain confined and may regress over time (Ogawa, 2017).

Histologically, hypertrophic scars are typically characterized by relatively organized, parallel collagen bundles, often with a more prominent type III collagen component, consistent with an exaggerated but partially self-limited repair response (Wolfram et al., 2009). By contrast, keloids exhibit dense, disorganized collagen bundles arranged in whorled or nodular patterns, frequently accompanied by mucin deposition and marked tissue stiffness (Limandjaja et al., 2020). These structural differences are consistent with more sustained fibroblast activation and impaired resolution of fibrotic signaling in keloids.

At the EV level, keloid fibroblast-derived vesicles have been reported to contain pro-fibrotic cargos such as miR-21, which suppress Smad7 and enhance TGF-β signaling (Yan et al., 2020). In contrast, hypertrophic scar-derived vesicles have been associated with inflammatory signaling pathways such as TAK1/NF-κB (Sakurai, 2012). This may suggest a more context-dependent integration of inflammatory and fibrogenic cues in hypertrophic scars. However, the comparative evidence base remains relatively limited, and many reported differences are drawn from separate model systems rather than direct head-to-head analyses. Therefore, EV-associated distinctions between keloids and hypertrophic scars are biologically plausible and potentially important, but should still be interpreted cautiously Table 1.

**TABLE 1:** Comparison of keloids and hypertrophic scars from clinical, histological, and exosome-mediated perspectives.

Feature	Hypertrophic scars	Keloids
Clinical behavior	Confined to wound margins; may regress	Extend beyond wound margins; rarely regress
Recurrence risk	Variable/Moderate	Generally high
Collagen organization	Relatively organized, parallel bundles	Dense, disorganized, whorled/nodular bundles
Collagen composition	Relatively higher type III proportion	Higher type I with type III; disordered deposition
ECM stiffness	Increased (moderate)	Increased (often marked)
Dominant fibroblast phenotype	Activated but more transient/resolution-prone	Sustained activation; apoptosis-resistant; invasive-like features (reported)
Exosomal miRNA profile	Heterogeneous/context-dependent; less consistent pro-fibrotic enrichment	Frequently reported enrichment of pro-fibrotic miRNAs (e.g., miR-21)
Key signaling pathways	Inflammation–fibrosis integration signals (e.g., TAK1/NF-κB), context-dependent	Sustained pro-fibrotic signaling (TGF-β/Smad; often with PI3K/Akt/Wnt co-activation)
Fibrosis outcome	Partial resolution possible	Progressive and refractory

To provide a clearer overview of the current evidence, representative studies of EV-/sEV-mediated communication in pathological scarring are summarized in Table 2, including vesicle source, cargo composition, experimental model, functional outcomes, and strength of evidence.

**TABLE 2:** Representative studies of EVs/sEVs in pathological scarring: source, cargo, model, and functional outcomes.

Study	Model/Evidence level	Vesicle source	Reported cargo/pathway	Main functional outcome	Interpretation/Methodological note
Li Q. et al. (2021)	Keloid fibroblasts; mainly *in vitro*	Keloid fibroblast-derived vesicles, described as exosomes	miR-21; SMAD7 suppression; TGF-β/Smad activation	Promoted fibroblast proliferation and collagen production	Mechanistically relevant and closer to causal evidence because a specific cargo-pathway axis was tested, but still largely cell-culture based and should be interpreted as preclinical evidence
Cui et al. (2022)	Hypertrophic scar fibroblasts and normal dermal fibroblasts; *in vitro*	Hypertrophic scar fibroblast-derived vesicles, described as exosomes	Smad and TAK1 signaling	Induced pro-fibrotic signaling in normal dermal fibroblasts	Supports fibroblast-to-fibroblast pathogenic crosstalk, but evidence remains mainly *in vitro* and does not fully establish *in vivo* necessity
Zhao et al. (2022)	Pathological scar-related fibroblast assays; *in vitro*/preclinical	MSC-derived vesicles	miR-138-5p targeting SIRT1	Inhibited fibroblast proliferation, migration, and fibrosis-related protein expression	Suggests an anti-fibrotic therapeutic mechanism, but translational value is limited by preclinical design and source/manufacturing variability
Yuan et al. (2021)	Excessive scar model; animal + *in vitro*	miR-29a-modified ADSC-derived vesicles	miR-29a; TGF-β2/Smad3 inhibition	Reduced excessive scar formation and collagen deposition	Stronger than correlation because cargo modification was tested functionally; still a preclinical proof-of-concept
Zhen et al. (2025)	Hypertrophic scar fibroblasts and rat tail scar model; *in vitro* + animal	Epidermal stem cell-derived EVs	miR-200 family; ZEB1/2	Induced mesenchymal–epidermal transition and alleviated hypertrophic scarring	A relatively strong mechanistic study because it combined cargo analysis with functional validation *in vivo*
Zhao et al. (2025)	Hypertrophic scar-derived fibroblasts and animal model; *in vitro* + animal	Epidermal stem cell-derived EVs	miR-203a-3p; PIK3CA; PI3K/AKT/mTOR	Promoted myofibroblast dedifferentiation and reduced scarring	Provides relatively direct cargo-pathway-function linkage, although still preclinical
Wang et al. (2017)	Cutaneous wound/scarless repair model; animal + *in vitro*	Human adipose MSC-derived vesicles	ECM-remodeling-associated cargos	Promoted scarless repair and ECM remodeling	Important for therapeutic narrative, but not specific to pathological scar causation in humans
Zhang et al. (2015)	Cutaneous wound model; animal + *in vitro*	iPSC-MSC-derived vesicles	Pro-regenerative cargos; angiogenesis-related signaling	Enhanced wound healing, collagen synthesis, and angiogenesis	Useful supportive evidence for regenerative EV therapy, though not specific to keloid/HS pathology
Ma et al. (2019)	Cutaneous wound healing model; animal + *in vitro*	ADSC-derived vesicles	Wnt/β-catenin-related signaling	Promoted proliferation and migration during wound healing	Demonstrates pathway relevance, but pro-healing effects in wounds should not be assumed to equal anti-scar effects in pathological fibrosis
Qian et al. (2021)	Cutaneous repair model; animal + *in vitro*	ADSC-derived vesicles	lncRNA H19/miR-19b/SOX9 axis	Accelerated wound healing	Supports the importance of lncRNA cargo, but whether H19 is anti-fibrotic or simply pro-repair is context dependent
Bo et al. (2022)	Burn wound healing model; animal + *in vitro*	iPSC-derived keratinocyte vesicles	miR-762	Promoted keratinocyte and endothelial migration	Supports epidermal/vascular communication, but evidence for direct relevance to keloids or hypertrophic scars remains indirect
Yan et al. (2025)	Mouse wound healing and diabetic wound model; animal + *in vitro*	Fibroblast-derived vesicles	miR-29a-3p; KEAP1/Nrf2	Improved wound healing quality and oxidative stress responses	Supports fibroblast-derived EV bioactivity, but not necessarily pathogenic fibroblast EV signaling in pathological scars
Zhong et al. (2023)	Review-level evidence	Multiple MSC sources	Multiple cargos and pathways	Summarized therapeutic potential in keloids and hypertrophic scars	Useful as synthesis, but not primary mechanistic evidence
Welsh et al. (2024)	Methodological guideline	EV field broadly	MISEV2023 nomenclature, separation, characterization standards	Recommends using “exosome” only with demonstrated endosomal origin; otherwise EV/sEV terminology is preferred	Essential for interpreting scar literature because many “exosome” studies may more accurately represent sEV-enriched EV preparations
Takakura et al. (2024)	Translational/regulatory review	Therapeutic EV products broadly	Manufacturing, QC, safety, potency, regulatory issues	Highlights scale-up, batch heterogeneity, safety, and release testing challenges	Useful for strengthening the translational discussion section
Xia et al. (2024)	Translational/safety review	Therapeutic EVs broadly	Immunogenicity and clearance determinants	Notes that EVs may not be immunologically inert	Important caution for therapeutic EV claims in scar treatment

## 4 EV-/sEV-associated signaling networks in pathological scarring

### 4.1 TGF-β/Smad signaling axis

The TGF-β/Smad pathway is widely regarded as a central driver of fibroblast-to-myofibroblast transition and excessive extracellular matrix accumulation in cutaneous fibrosis. Increasing evidence suggests that EV-/sEV-associated cargos, particularly non-coding RNAs, may modulate this pathway in pathological scarring. Among the most frequently reported examples, miR-21 has been associated with suppression of SMAD7, a negative regulator of TGF-β signaling, thereby promoting Smad2/3 phosphorylation and increasing the expression of pro-fibrotic genes, including collagen and α-SMA-related programs (Saa et al., 2021; Yousefi et al., 2020). In contrast, miR-29 family members, including miR-29a, are generally considered anti-fibrotic regulators that suppress collagen synthesis and other ECM-related genes. Reduced miR-29 expression has therefore been linked to ECM imbalance and fibrotic progression (Pan et al., 2024). Together, these observations suggest that shifts in vesicle-borne miRNA programs can bias TGF-β/Smad signaling toward persistent activation and thereby contribute to pathological scar formation.

### 4.2 Wnt/β-catenin and PI3K/Akt pathways

In addition to TGF-β/Smad signaling, EV-/sEV-associated cargos have also been implicated in Wnt/β-catenin and PI3K/Akt pathways. These pathways regulate fibroblast proliferation, survival, and ECM production, making them plausible contributors to pathological scarring.

Vesicles carrying Wnt-related regulators, including miRNAs such as miR-130b, have been reported to promote β-catenin accumulation in fibroblasts, thereby enhancing fibrotic responses (Zhang et al., 2023b). Furthermore, Wnt and TGF-β pathways may interact synergistically to sustain myofibroblast activation. However, many studies rely on pathway enrichment analysis rather than direct manipulation of vesicle cargo, limiting mechanistic certainty.

Similarly, PI3K/Akt activation has been linked to EV-associated cargos that promote fibroblast survival and resistance to apoptosis (SMN Mydin et al., 2024). These effects may contribute to scar persistence and recurrence. However, the functional consequences of PI3K/Akt signaling may vary depending on vesicle source, cargo composition, and microenvironmental context.

Overall, while Wnt/β-catenin and PI3K/Akt pathways are plausible EV-responsive signaling networks, the available evidence remains less robust than that for TGF-β/Smad signaling.

### 4.3 NF-κB and inflammatory signaling

NF-κB is a central regulator of inflammatory responses and fibrosis. Immune cell-derived EVs have been implicated in modulating NF-κB signaling in scar tissue. Vesicles derived from M1-like macrophages have been associated with pro-inflammatory cargos, including miR-155, which may activate NF-κB signaling and increase expression of inflammatory mediators such as IL-6 and COX-2 (Xu et al., 2023). Conversely, MSC-derived EVs carrying miR-146a have been reported to suppress NF-κB activation and promote anti-inflammatory remodeling (Chen et al., 2021). Because NF-κB signaling interacts with TGF-β pathways, vesicle-mediated modulation of inflammation may indirectly influence fibrotic progression.

However, many studies infer NF-κB involvement based on downstream gene expression changes rather than direct pathway interrogation. Therefore, causal relationships between EV cargo and NF-κB activation remain incompletely defined.

### 4.4 Non-coding RNAs in exosome-mediated crosstalk

Non-coding RNAs are among the most extensively studied EV-associated cargos in pathological scarring. These molecules regulate multiple signaling pathways involved in fibroblast activation, inflammation, and ECM remodeling. miR-21 is consistently reported as a pro-fibrotic regulator, whereas miR-29 family members are associated with anti-fibrotic effects (Floris et al., 2025; Lucaciu et al., 2025). MiR-200b has also been implicated in epithelial plasticity and EMT-associated processes (Han et al., 2020). Long non-coding RNAs, such as H19, may function as competing endogenous RNAs, modulating miRNA activity and downstream fibrotic signaling (Yan et al., 2021). CircRNAs represent an emerging regulatory layer, although evidence for circRNA-mediated effects in pathological scarring remains limited (Dragomir et al., 2018).

**FIGURE 1:**
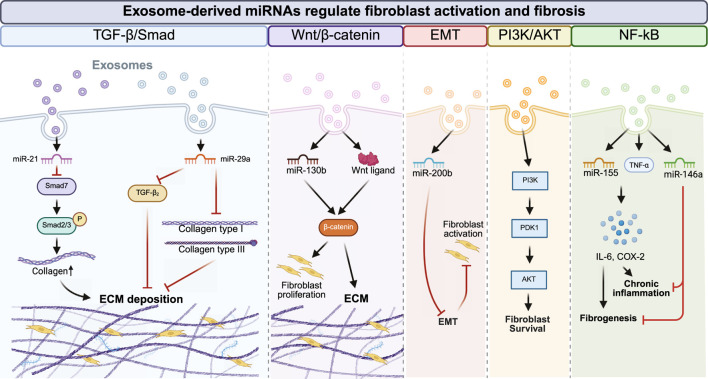
Exosome-derived miRNAs regulate fibroblast activation and fibrosis. Schematic showing exosome-associated cargos modulating key pathways in pathological scarring. miR-21 enhances TGF-β/Smad signaling via Smad7 suppression, promoting collagen/ECM deposition, whereas miR-29a counteracts fibrosis by inhibiting TGF-β2 and collagen expression. miR-130b and Wnt-associated signals activate Wnt/β-catenin to drive fibroblast proliferation/ECM production. miR-200b restrains EMT and fibroblast activation. Exosomal components activate PI3K–PDK1–AKT to support fibroblast survival. Inflammation-related cargos (e.g., miR-155, TNF-α) stimulate NF-κB (IL-6/COX-2), while miR-146a exerts inhibitory effects. Abbreviations: ECM, extracellular matrix; EMT, epithelial–mesenchymal transition.

An important unresolved question is whether EV cargo changes represent causal drivers of fibrosis or secondary consequences of donor-cell activation. Figure 1 Many studies report altered ncRNA expression without demonstrating functional necessity or sufficiency. Therefore, EV-associated ncRNAs should currently be considered promising but still incompletely validated regulators of pathological scarring.

## 5 Therapeutic perspectives: from EV biology to translational strategies

### 5.1 Targeting EV-/sEV-associated signaling pathways

Aberrant EV-/sEV-mediated signaling is increasingly implicated in sustaining fibroblast activation, inflammation, and extracellular matrix overproduction in keloids and hypertrophic scars. One mechanistically attractive strategy is to disrupt pathological intercellular communication by inhibiting vesicle biogenesis, cargo loading, release, or uptake. For example, GW4869, an inhibitor of neutral sphingomyelinase 2 (nSMase2), has been reported to suppress the release of ceramide-dependent sEV-enriched vesicle populations and to attenuate pro-fibrotic signaling outputs, including TGF-β- and PI3K/Akt-associated programs, in experimental scar-related settings (Zhong et al., 2023). These findings suggest that interference with vesicle release may help interrupt self-reinforcing fibrotic signaling loops.

However, this strategy remains largely experimental. Broad inhibition of EV secretion may impair not only pathogenic signaling but also physiological vesicle-mediated functions required for normal wound healing, immune regulation, and tissue homeostasis. In addition, current inhibitors are not vesicle-source specific and may affect multiple cellular processes beyond EV biogenesis. Questions regarding specificity, dose optimization, delivery route, tissue selectivity, and off-target toxicity remain insufficiently resolved. Therefore, inhibition of EV biogenesis or release should currently be regarded as a promising but still incompletely validated antifibrotic strategy rather than a near-term therapeutic option.

### 5.2 EV-/sEV-based therapeutics

EV-/sEV-enriched preparations derived from MSCs are widely explored as potential cell-free therapeutics because they may convey anti-inflammatory and anti-fibrotic signals while supporting regenerative repair. Reported MSC-derived cargos include regulatory miRNAs, such as members of the miR-29 family, together with proteins and other bioactive molecules that may suppress fibroblast activation, dampen pro-fibrotic signaling, and limit excessive ECM accumulation (Yu et al., 2024). In addition to their native effects, such vesicles may also be engineered to deliver therapeutic payloads, including siRNAs, synthetic miRNAs, or pathway-specific inhibitors, with the goal of improving potency and target selectivity.

In preclinical studies, engineered EVs targeting Smad- and PI3K/Akt-associated programs have been reported to suppress fibroblast-to-myofibroblast transition and reduce scar burden in animal models (Jibing et al., 2024). Biomaterial-based delivery systems, such as GelMA-derived hydrogels loaded with vesicle preparations, may further improve local retention and sustained release, thereby enhancing modulation of collagen deposition, vascular remodeling, and inflammatory responses in hypertrophic scar models (Wang et al., 2025). These findings support the therapeutic potential of EV-based approaches; however, current evidence remains overwhelmingly preclinical, and the precise active cargos, dose–response relationships, pharmacokinetics, and long-term effects remain incompletely defined.

### 5.3 Combination therapies and translational considerations

Given the limitations of monotherapy, combination strategies integrating EV-/sEV-based interventions with established scar treatments are attracting increasing interest. For example, combining MSC-derived vesicles with fractional laser resurfacing, corticosteroid-based approaches, or antifibrotic agents such as pirfenidone may improve scar pliability and remodeling by targeting both ECM turnover and the inflammatory microenvironment (Murakami and Shigeki, 2024). In parallel, multifunctional biomaterials capable of co-delivering EVs together with small-molecule inhibitors may provide spatially controlled and sustained modulation of fibrosis-related processes, including inflammation, angiogenesis, and matrix deposition (Zhong et al., 2024). Such approaches are conceptually attractive because pathological scarring is driven by multiple interacting mechanisms rather than a single pathway. Combination regimens may therefore be particularly relevant in refractory keloids, where persistent pro-fibrotic signaling and high recurrence rates remain major barriers to durable control. However, evidence for therapeutic synergy remains limited, and optimal treatment sequencing, dose selection, administration route, and long-term safety remain uncertain.

## 6 Conclusion

Pathological scarring, including keloids and hypertrophic scars, remains a major therapeutic challenge characterized by persistent fibroblast activation, dysregulated extracellular matrix remodeling, chronic inflammation, and aberrant intercellular communication. Increasing evidence suggests that extracellular vesicles (EVs), particularly small extracellular vesicles (sEVs), participate in these processes by modulating fibroblast phenotype, immune responses, angiogenesis, and epithelial–mesenchymal crosstalk.

Current studies indicate that vesicles released from fibroblasts, keratinocytes, endothelial cells, immune cells, and mesenchymal stromal/stem cells may contribute to pathological scar formation through signaling pathways such as TGF-β/Smad, Wnt/β-catenin, PI3K/Akt, and NF-κB. However, this literature should be interpreted cautiously. Much of the available evidence is derived from *in vitro* experiments or animal models, whereas direct clinical evidence remains limited. In addition, many reported mechanisms are based on cargo profiling, pathway enrichment, or association analyses rather than on direct causal experiments demonstrating that a defined vesicle cargo is necessary and sufficient to drive fibrosis.

From a translational perspective, EV-based approaches, including inhibition of vesicle release, MSC-derived vesicle preparations, engineered EVs, and biomaterial-assisted delivery systems, are promising but remain investigational. Major barriers to clinical application include inconsistent vesicle nomenclature, methodological heterogeneity in isolation and characterization, incomplete standardization of potency and dose metrics, batch-to-batch variability, storage instability, uncertain biodistribution and targeting, and unresolved safety and regulatory issues. An additional unresolved question is whether altered EV cargo is a primary driver of pathological scarring or a secondary consequence of donor-cell activation and the fibrotic microenvironment.

Future studies should therefore prioritize rigorous alignment with MISEV recommendations, clearer distinction between exosomes and other EV subtypes, standardized isolation and characterization workflows, and stronger causal validation across *in vitro*, animal, and clinical settings. A more critical and methodologically robust understanding of EV-/sEV-mediated communication may help advance both biomarker development and the rational design of targeted therapies for pathological scarring.
